# Peripheral and placental immune responses in sheep after experimental infection with *Toxoplasma gondii* at the three terms of gestation

**DOI:** 10.1186/s13567-019-0681-8

**Published:** 2019-09-18

**Authors:** Pablo Castaño, Miguel Fernández, Javier Regidor-Cerrillo, Miguel Fuertes, Pilar Horcajo, Ignacio Ferre, M. Carmen Ferreras, Luis Miguel Ortega-Mora, Valentín Pérez, Julio Benavides

**Affiliations:** 1 Departamento de Sanidad Animal, Instituto de Ganadería de Montaña (CSIC-ULE), Grulleros, 24346 León, Spain; 2 SALUVET, Animal Health Department, Faculty of Veterinary Sciences, Complutense University of Madrid, Ciudad Universitaria s/n, 28040 Madrid, Spain

## Abstract

Although it is known that gestation could influence the clinical course of ovine toxoplasmosis, the precise effect of the term of gestation when sheep are infected are yet mostly unknown. The aim of this study was to evaluate the peripheral and placental immune responses developed in pregnant sheep after experimental infection with *Toxoplasma gondii* at different times of gestation. Thirty-six pregnant sheep were allocated in different groups, orally inoculated with sporulated oocysts of *T. gondii* at early, mid and late gestation and culled within 30 days post-infection. The peripheral humoral and cytokine responses were evaluated, as well as the transcription of cytokines at the placenta. Serological analysis revealed that, regardless the term of gestation when infected, specific IgG against *T. gondii* were detected from day 8 post-infection and there was an early peripheral release of IFN-γ at the first week post-infection followed by a short peak of IL10 and TNF-α at the second week post-infection. There were no significant differences in this response between infected groups. At the placenta, a similar increase in transcription of IFN-γ, and TNF-α was found at the three terms of gestation, while IL-4 increased mainly at the first and second terms and IL-10 transcription was higher at the last term. While these findings show that both Th1 and Th2 cytokines play a key role in the pathogenesis of ovine toxoplasmosis and that placental and peripheral immune responses do not closely correlate, there seems to be no clear modulation of these responses along the gestation.

## Introduction

Ovine toxoplasmosis is an important infectious disease, caused by the protozoan *Toxoplasma gondii*, that results in heavy economic losses in the sheep industry worldwide as it is related to reproductive failure, principally abortions and weak newborn lambs [1]. Despite the great importance of this disease little is known about the mechanisms underlying the abortions, as it is not clear whether the cause of abortion is a direct consequence of the parasite multiplication in the foetus or the placenta, or, it is caused by a deregulation of hormones or immune responses in this tissue [2].

There is clear evidence that the consequences of ovine toxoplasmosis heavily depend on the term of gestation when infection occurs [2]. Although there is clear evidence that the placenta is under immunomodulation during pregnancy and that placental and peripheral immune responses differ at this time [3], this premise has not yet been proven to occur in pregnant sheep [4]. The paradigm that maternal immune response at the placenta level shifts from a Th1 phenotype, characterized by IFN-γ and TNF-α production, towards a Th2 phenotype, mainly represented by IL4 and IL10 production, from mid gestation is mostly based on murine experimental models [5]. However, these evidence cannot be extrapolated to sheep as there are several differences between mice and sheep in the histological structure of placenta, immune response and duration of pregnancy [6]. In fact, the few studies carried out to investigate this paradigm in sheep have not found differences at the peripheral immune response between pregnant and non-pregnant ewes [7, 8].

The studies that focus on the immune response following infection of pregnant ewes with the protozoan *T. gondii* are scarce [9–11]. It seems clear that an early production of the pro-inflammatory cytokine IFN-γ is an important mechanism to control the infection by inducing a Th1 immune response [12, 13]. In addition to cellular mechanisms, *T. gondii* infection in ewes is known to stimulate humoral immune response as well [14], although it is not until the second week after infection when antibodies are detected in maternal peripheral blood [15] and they play a minor role in controlling the parasite [16]. On the other hand, it is well known the importance that the placenta has as an inductor of immunity to prevent foetus infections and to allow the normal course of pregnancy in ruminants [17]. However, despite the relevance of ovine toxoplasmosis, there are very few studies investigating the placental immune response developed during this disease. The influence of the time of gestation when infections occurs on the clinical course, development of lesions and parasite multiplication on ovine toxoplasmosis has been recently studied in an experimental model of pregnant sheep [2]. Bearing in mind the lack of evidence on immunomodulation at the peripheral level [8], we hypothesized that the placental immune response and its possible modulation during gestation play a key role in the pathogenesis of ovine toxoplasmosis. The present study is aimed to compare the placental and peripheral immune responses developed in pregnant sheep after oral infection with sporulated oocysts at the three terms of gestation. The samples for this study come from a previous study where the influence of gestation on the clinical course was shown [2].

## Materials and methods

### Animals and experimental design

A full description of the experimental design was described previously [2]. Thirty-six pure Churra breed primiparous sheep aged 24–30 months, seronegative for *T. gondii*, *Neospora caninum*, border disease virus, *Coxiella burnetii* and *Chlamydia abortus* were oestrus synchronized and mated with pure breed Churra tups for 2 days, after which the rams were removed from the ewes. Pregnancy and foetal viability were confirmed by ultrasound scanning (US) on day 40 after mating and again before infection. The pregnant sheep were randomly distributed into three experimental groups, each of them formed by 9 infected sheep and 3 negative, non-infected, control sheep.

Depending on the term of gestation when infected, sheep were allocated into Group 1 (*n* = 12, G1), inoculated at day 40 of gestation; Group 2 (*n* = 12, G2), inoculated at day 90 of gestation, and Group 3 (*n* = 12, G3), inoculated at day 120 of gestation (Table 1). Nine sheep from each three groups were orally inoculated with 50 sporulated oocysts of the M4 isolate of *T. gondii* (Moredun Research Institute, Edinburgh, Scotland, UK), a Type II isolate (Dr Frank Katzer, personal communication) diluted in 50 mL of PBS, whereas the 3 control sheep of each group received 50 mL of PBS as negative control of inoculation. The experiment was designed in order to cull four sheep, three infected sheep and one control of each group, at 12, 19 and 26 days post-infection (dpi) or when foetal death was observed at the US scan or the sheep delivered a stillbirth. However, due to the occurrence of early (i.e. between 9 and 16 dpi) and late (between 17 and 26 dpi) spontaneous abortions, the number of infected ewes culled on days 12, 19 and 26 pi for G2 and G3 had to be modified with regard to the initial experimental design (Table 1).

**Table 1:** **Experimental design and clinical outcome of experimental animals according to the day of gestation when infected: day 40 (G1), day 90 (G2) and das 120 (G3) and the day post-infection (dpi) when ewes were culled or abortion occurred**

	Number of sheep				
	Foetal death/stillbirths^a^		(Serial culling)		
	6–16 dpi	17–26 dpi	12 dpi	19 dpi	26 dpi
G1 (*n* = 12)	2/0^b^	1/0^b^	1/1^b^	3/1^b^	2/1^b^
G2 (*n* = 12)	4/0	1/0	1/1	2/1	1/1
G3 (*n* = 12)	2/0	4/0	2/1	1/1	0/1

^a^Spontaneous abortions or stillbirths occurring in that group, the other timepoints (12, 19 and 26 dpi) correspond to serials culling of pregnant ewes.

^b^Infected/control sheep.

This experiment was carried out according to the Guidelines of the European Union Council (2010/63/EU) for the use of laboratory animals and was in accordance with local national guidelines (RD 53/2013) which regulates the welfare of animals used for experimentation. They were also approved by the CSIC bioethics committee (OH416-2016).

### Pregnancy monitoring and sample collection

Once inoculated, foetal viability was determined by monitoring the heartbeat trough US scan 3 days a week.

Blood samples were collected at −1, 1, 5, 8, 12, 15, 19, 22 and 26 dpi by jugular veni-puncture and at the day of culling from the dams before euthanasia and from umbilical cord veins or heart during necropsy from the foetuses. These samples were collected into Vacutainer tubes (Becton–Dickinson and Company, Plymouth, UK) without anticoagulant and allowed to clot. Serum was obtained by centrifugation and samples were stored at −80 °C until analysis.

At 12, 19 and 26 dpi, or after spontaneous abortions (see chapter above) sheep were sedated with xylazine (Rompun™, Bayer, Mannhein, Germany) and then immediately euthanized by an intravenous overdose of embutramide and mebezonium iodide (T61, Intervet, Salamanca, Spain). Immediately after euthanasia, both placental and foetal tissues were selected and recovered. Ten randomly selected placentomes recovered from each placenta were transversally cut and immersed in RNAlater (Sigma-Aldrich, Saint Louis, MO, USA) for cytokine mRNA expression analysis.

### Detection of specific anti-*T. gondii* IgG in sera

Serum recovered from all the ewes and foetuses were used in an in-house indirect ELISA [15] to measure specific anti-*T. gondii* antibodies. Soluble antigen from tachyzoites of ME49 isolate was used to coat 96 well microtiter plates. Briefly, 100 μL/well of antigen solution at 10 μg/mL diluted in carbonate buffer (63 mM, pH 9.6) was incubated overnight at 4 °C. Subsequently, non-specific binding was blocked by adding 100 μL of bovine serum albumin diluted 0.05% in phosphate buffer saline (0.1 M, pH 7.6) containing 0.05% Tween 80 (PBST). After 2 h incubation at room temperature, the plates were washed four times with PBST. Sera samples were diluted 1:100 in PBST and 100 μL of this dilution was added to each well and incubated for 60 min at room temperature. All samples were analyzed in duplicate. After four washes in PBST, 100 μL of horseradish peroxidase conjugate protein G (Biorad, Hercules, USA) diluted 1:1500 in PBST was added as secondary antibody (Ab) and incubated for 1 h at room temperature. After washing again, 100 μL per well of substrate ABTS (Sigma-Aldrich, Madrid, Spain) diluted 5.48 mg in 50 mL of citrate buffer 0.05 M, pH 4.0 with 0.0016% hydrogen peroxide was added to each well. Finally, after 30 min of incubation, 40 μL per well of a solution of hydrofluoric acid 0.1 M was at room temperature to stop the reaction and the optical density of each well (OD) was read at 405 nm (OD405). The results were given as an optical index (OI) of the OD ration value (OI = [OD sample − OD negative control]/[OD positive control − OD negative control]).

### Determination of IFN-γ, TNF-α, IL4 and IL10 levels in maternal sera

The levels of IFN-γ and IL4 cytokines were measured using the Bovine IFN-γ and IL4 ELISA development kits (Mabtech, AB, Sweden), following the kit manual instructions. On the other hand, TNF-α and IL10 assays were performed using the specific Cusabio^®^ competitive inhibition ELISA for each cytokine, according to manufacturer’s recommendations. In all cases, plates were read at 450 nm and the results were interpreted using the “Curve Expert Professional” software program (Hyams Development, AL, USA). In order to compare the evolution of the serological level of cytokines in each term of gestation, data from 1, 5, 8, 12, 15, 19, 22 and 26 dpi were normalized to the −1 dpi value of each group and expressed as a ratio.

### RNA extraction and quantitative real-time PCR (qPCR)

RNA was extracted from five random placentomes using the commercial Maxwell^®^ 16 LEV simplyRNA Purification Kit, developed for automated Maxwell^®^ 16 System (Promega, Wisconsin, USA), following the manufacturer’s recommendations. RNA integrity was checked by 1% agarose gel and RNA concentrations were determined using the spectrophotometer Nanophotometer (Implen GmbH, MU, GER). cDNA was obtained by reverse transcription using the master mix SuperScript^®^ VILO™ cDNA Synthesis Kit (Invitrogen, Paisley, UK) following the procedures described previously [16]. Primers and real-time PCR reactions (Additional file 1) were performed, as described previously by Arranz-Solis et al. [18]. The mRNA expression was calculated normalizing the Ct value of the target gene with the Ct value of the housekeeping gen. Normalized Ct values from infected animals were subtracted from average Ct values obtained from control animals. The resulting differences (ΔΔCt) were transformed to, and express as, linear fold increase regarding the control animals and subjected to statistical analysis, as described previously [18, 19].

### Statistical analysis

Given the limited group sample sizes after the initial culling schedule had to be modified because of the occurrence of early and late spontaneous abortions, the effect of the day post-infection when sheep was culled or aborted were assumed to be non-significant and in each group data were pooled in three categories according to the time of gestation when ewes were infected (G1, G2 and G3).

Antibody responses for each experimental group were analyzed using the one-way ANOVA test. When statistically significant differences were found, a Tukey’s Multiple Range test was applied to examine all possible pairwise comparisons at each sampling time. Variations in serological cytokine levels from sera were analyzed by multiple t tests, using the Holm–Sidak method to establish a correction for multiple comparisons.

Finally, to assess differences between each infected group a Mann–Whitney test was performed on cytokine mRNA expression levels in placenta.

In all analysis, statistical significance was established in *P* < 0.05. GraphPad Prism 6.01 software (San Diego, CA, USA) was employed to analyze all the data.

## Results

Detailed results of the clinical course, lesional development and parasite distribution were published in Castaño et al. [2]. Additional file 2 shows a brief comparison of the parasite burden and histological score values of infected ewes from the three groups (G1, G2 and G3) studied at 26 dpi.

### Specific serological anti-*T. gondii* IgG antibody response

**Figure 1:**
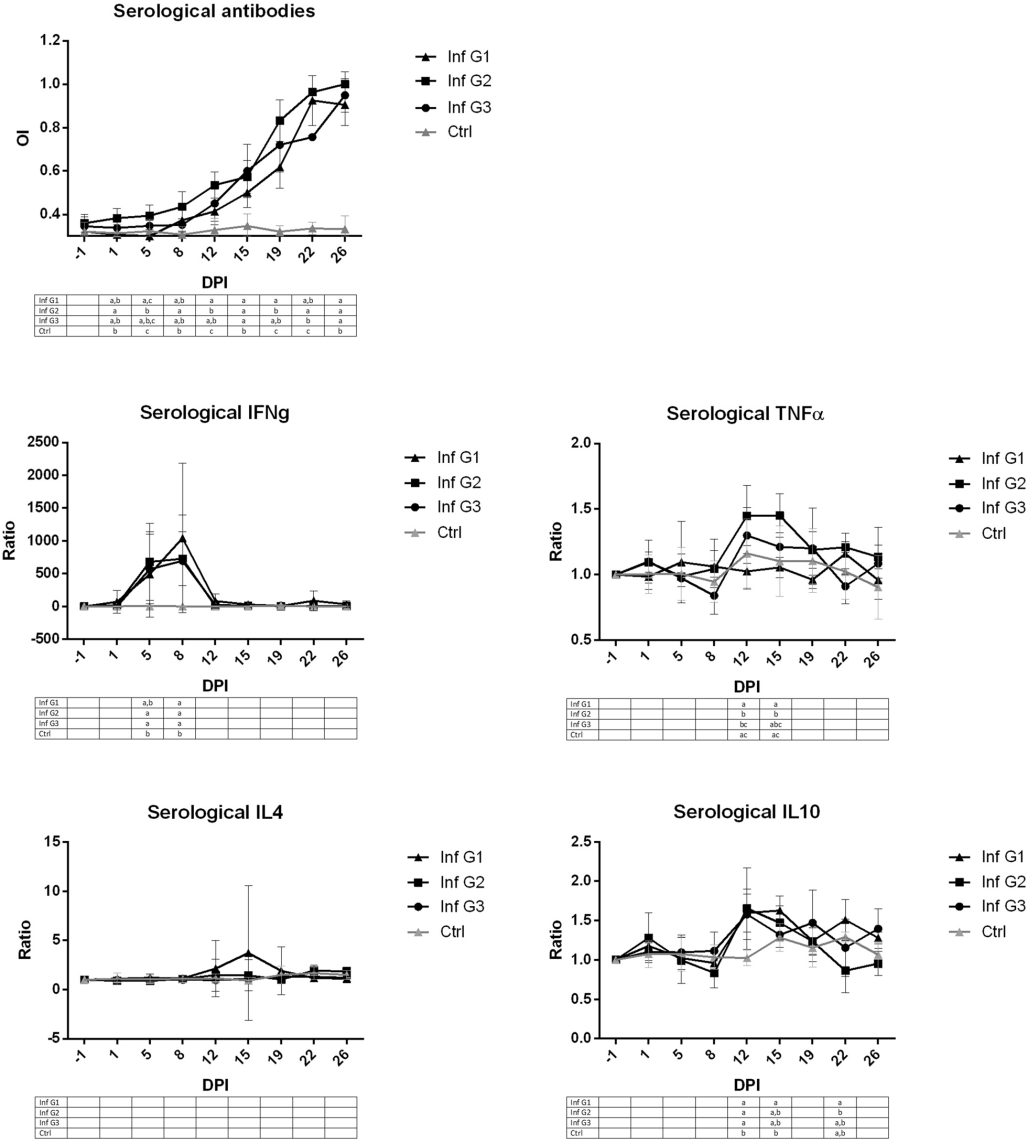
**Antibody and cytokine production in sera from the three infected groups and control ewes.** Graphs representing optical index (OI) of antibodies and cytokines ratio of infected ewes at early (Inf G1), mid (Inf G2) and late (Inf G3) gestation as well as in non-infected animals (Ctrl) to analyse the influence of the gestation term when infected with the protozoan *T. gondii* occurred on the peripheral immune response. Different letters in the same column in each table beneath the graphics indicate statistical differences (*P* < 0.05) between groups when comparing then at the same dpi.

Specific serological antibodies in the three infected groups were detected from day 5 post-infection (pi) and increased until the end of the experiment. While the pattern of serological antibodies was very similar among the three groups, those ewes infected at mid gestation (G2) showed significantly higher OI than those animals infected at early gestation, specifically on days 1, 5, 8 and 12 pi, and those infected at late gestation, specifically on days 8, 12, 19 and 22. All control animals showed basal levels throughout the study (Figure 1).

### Cytokine kinetics in maternal sera

The strongest and more evident response, also the earliest, measured by ELISA and found in maternal sera was that of IFN-γ (Figure 1). While the three infected groups showed significant increase of serological IFN-γ at day 8 pi when compared to control animals, G2 and G3 infected sheep also showed higher levels than control at day 5 pi. There were no statistically significant differences between infected groups. On the other hand, the increase of TNF-α at day 12 dpi was not the same in the three groups as this increase was mainly found in G2, which had significantly higher level than controls and G1 at days 12 and 15 pi. Regarding IL10, there were no statistically significant differences between infected groups at day 12 pi, when all of them showed higher level than control animals, although G1 levels were higher than G2 at day 22 pi. G1 showed higher level of cytokine than controls at day 19 pi. There were no significant differences between cytokine levels at any of the three infected groups or control animals regarding IL4 (Figure 1).

### Cytokine RNA expression at the placenta

**Figure 2:**
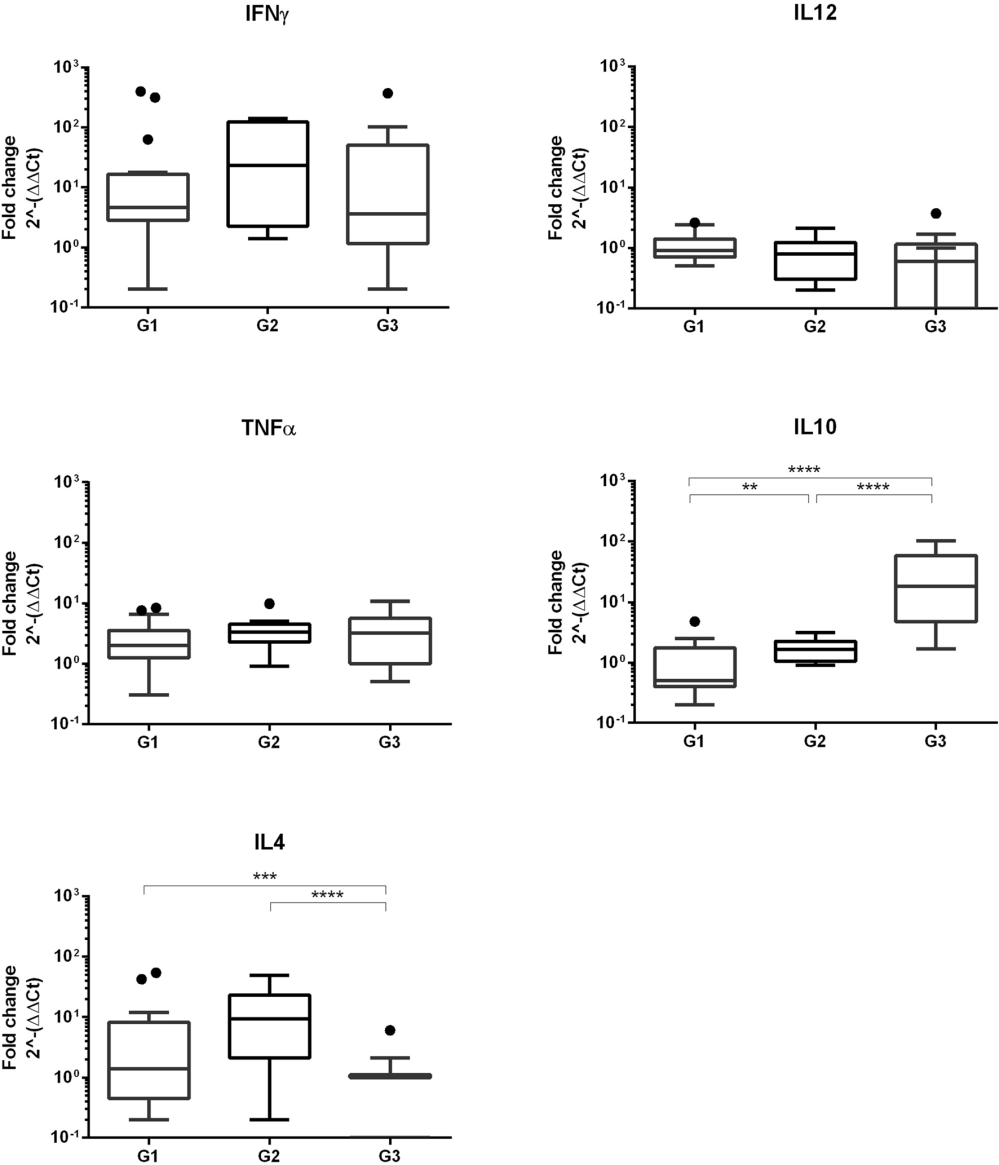
**Cytokine transcription at the ovine placentomes of the three infected groups and control ewes.** Graphs representing Th1 (IFN-γ and TNF-α) and Th2 (IL4 and IL10) cytokine transcription at the placentomes from infected ewes at early (Inf G1), mid (Inf G2) and late (Inf G3) gestation in comparison with the non-infected ewes of their respective groups (Ctrl 1, Ctrl 2 and Ctrl 3) to analyse the influence of the gestation term when infected with the protozoan *T. gondii* occurred on the placental immune response.

The immune response at the placenta was characterized by analyzing the RNA transcription of IFN-γ, TNF-α, IL4, IL10 and IL12 at the placentomes (Figure 2). All the cytokines except IL12 showed significant increase of fold change in at least one term of gestation when compared to the control, non-infected animals. Regarding those that increased only at certain terms, fold change increase in IL10 was not significantly different to the control animals at the first term of gestation (G1), while in IL4 the loss of significance occurred on the third term (G3).

Analyzing those results when infected animals showed significant differences with the control sheep, and similarly as found in the serological cytokines, the highest fold change was found in IFN-γ at the three times of gestation. When comparing between the groups, no significant differences were found among them, so the transcription of IFN-γ was similar regardless the time of gestation when infection occurred. Similarly, the increase of the transcription in TNF-α was similar among the three groups.

When analyzing Th2 cytokine transcription levels at the placentomes, besides differences with their control animals, there were also differences between terms of gestation. While IL4 was significantly higher at the first and second term, IL10 was precisely higher at the last term.

## Discussion

We have previously reported the clear influence of the time of gestation when sheep are infected on the pathogenesis of experimental ovine toxoplasmosis, when sheep infected at the third term of gestation showed the earlier occurrence of lesions and colonization of the placenta by the parasite (day 19 pi vs. day 26 at the second and first term). However, the most severe lesions and higher burden of the parasite were found at 26 dpi after infection at mid gestation [2]. In order to gain insight into the pathogenesis of this disease, the objective of this work was to further characterize the immune response induced in pregnant ewes, both peripherally and placentally.

When analyzing the humoral immune response, all infected groups showed higher levels of specific antibodies than control group from day 12 pi. The kinetic of the serological antibodies was similar regardless the time of gestation when infected, although G2, infected at mid-gestation, had statistically significant (*P* < 0.0001) higher levels of antibodies than G1 on days 12 and 19 pi, but the level of antibodies become similar for the three groups after that and until the end of the experiment. This pattern of serological antibodies, rising from the second week post-infection is similar to that reported in previous experimental infections [10, 11, 20], and the similar results between groups suggest that the term of gestation when infection occurs does not influence significantly on the peripheral humoral immune response.

Regarding the serological cytokines, the three groups again show a very similar pattern, especially regarding IFN-γ, of which there is a significant (*P* < 0.0001) increase in serum levels after infection as early as 5 and 8 dpi. These results show that IFN-γ is a key cytokine after *T. gondii* infection during gestation, similarly as previously shown in non-pregnant sheep [15, 21]. After this peak of serological IFN-γ, it decreases coinciding with a gradual increase of specific anti-*T. gondii* serological antibodies, TNF-α and IL10. The increase in these two cytokines respectively, has been previously described in human trophoblasts experimentally infected with *T. gondii* [22]. The comparison between groups shows that the significant differences between groups were not consistent along the days after infection, appearing only on specific days, with the exception of TNF-α, which was higher on those animals infected at mid-gestation (i.e. G2) than the control animals but also than those infected at early gestation (i.e. G1) on days 12 and 15 pi. Bearing in mind, as we reported previously [2], that the more severe lesions, both placental and foetal, but also the higher parasite burden were found in ewes infected at mid gestation, it is possible to suggest that the cytokines measured when studying the peripheral immune response developed in ewes infected at mid gestation (i.e. G2), serological TNF-α, as well as antibodies against *T. gondii* infection, might be formed by cytokines secreted both in the placenta but also in peripheral lymph nodes, and that it could be related in some ways to the severe damage observed in both placental and foetal tissues. There were no significant differences (*P* > 0.5) between control and infected groups, nor between infected groups either, regarding IL4 serum levels, which agrees with previous results in experimental murine models of *T. gondii* infection [23]. In view of these results it is possible to elucidate that, most probably, IL4 does not play a pivotal role in promoting the host peripheral immune response during infection with *T. gondii*.

The influence of the infection was also investigated at placental level, studying the cytokine transcript expression profile at the materno-foetal interface. An increase mRNA transcription for all the cytokines analyzed, except from IL12, was detected in the placenta of the infected animals with regard to the control group. This finding suggests that, similarly to the peripheral level, there is a mixed Th1 and Th2 type placental immune response after infection with *T. gondii* in pregnant ewes. A similar response has been shown to occur after infection with the close protozoan *Neospora caninum*, both in sheep [18] and cattle [24], suggesting that in both diseases, toxoplasmosis and neosporosis, both types of cytokines, Th1 and Th2, are involved in the response against the parasite, and that there is not a clear polarization of the immune response during gestation. When analysing those Th1 cytokines which increased after the infection (i.e. TNF-α and IFN-γ), there are no statistically significant differences between groups. IFN-γ is the one that shows the highest fold increase in the three groups, suggesting again its importance not only at peripheral level but also at the placental level. The importance of this cytokine in the pathogenesis of ovine toxoplasmosis has been previously suggested [25, 26], and the current study shows that it plays a similar role regardless the term of gestation when the sheep is infected.

On the other hand, the pattern found in IL10 and IL4, are opposed. While IL10 increased after infection at the last term of gestation, IL4 showed higher levels on the first and second term. This behavior does not fit with the classical idea of the immune modulation paradigm during gestation, where Th2 cytokines, i.e. IL4, would be favored at the second half of gestation [5]. Our results show that there is not a clear tendency towards a Th2 preponderant immune response towards the end of gestation in pregnant ewes. On the contrary, it seems that IL4 mRNA levels at the foetal–meternal interface were higher at first and second term of gestation. Other studies analyzing the transcription of this cytokine after infection by the closely related protozoan, *N. caninum,* found an increase in IL4 levels in the placenta from sheep [18] and cattle [24] and even mice [27], confirming that this cytokine plays a key role in the pathogenesis of neosporosis. Specifically, in pregnant sheep, the level of IL4 mRNA after infection with *N. caninum*, was the same at the first and third terms of gestation [18]. Taken together the results from TNF-α, IFN-γ and IL4, both peripherally and at the placental level suggest that there is not a clear modulation of the immune response in pregnant sheep, at least under the circumstances of these studies. A previous study using a nominal antigen (i.e. not an infectious antigen), found no differences either in the production of different cytokines by peripheral blood mononuclear cells along the gestation in sheep immunized with chicken egg albumin as antigen (i.e. ovoalbumin) [8]. Although comparisons between this study and ours are difficult due to the different nature of the challenging stimuli (a nominal antigen, ovoalbumin, vs. a life protozoan, *T. gondii*).

On the other hand, there is a clear increase in the transcription of IL10 at the placenta at the second and third term of gestation. IL10 has been proposed as playing a key role in the maintenance of the gestation for its relation to regulatory T-cells [28]. Its role during *T. gondii* infection during pregnancy has not been deeply investigated, but recent studies in murine experimental models have shown that IL10 might be induced by *T. gondii* infection as an evasion strategy against the host immune response as a way to stablish chronic infection [29]. It is then tempting to hypothesize that, in the current experiment, the higher fold change in IL10 at the third term of gestation might have favored the earlier colonization of the placenta by the parasite, as observed in the previous study [2]. In this sense, and bearing in mind the results from IL4 and IL10, it is possible then that some modulation of the placental immune response might occur along gestation, but this modulation would not be orientated toward a Th2 response at the end of gestation. These results warrant further research into the modulation of the placental immune response in sheep and its role on the host response against infections.

However, when analyzing the behavior of these two cytokines, IL10 and IL4 at the peripheral levels and at the three terms of gestation, there is no correlation with the variations observed at the placenta. This would suggest that there are differences between those responses and that the host immune response against *T. gondii* infection might be different depending on the location. The fact that the immune response against *T. gondii* infection is modulated at the foetal–maternal interface, but not peripherally, has been previously documented in experimental *T. gondii* infections in mice [30], where regulatory T cells at the placenta express higher levels of CTLA-4 and PD-L1 than the same populations of cells at the spleen. Altogether, the findings from the current study suggest that, in sheep, there might be a subtle basal, and limited, modulation of the immune response in the placenta during gestation, similarly as described in other species, but this modulation may not reflect in changes at the peripheral level. The role of hormones on *T. gondii* infection has been widely discussed and several studies, based on murine or human cell lines, suggest that they could contribute to the modulation of *T. gondii* infection [31]. However, the only studies that analyzed the involvement of hormones in ovine toxoplasmosis were both carried out at the same term of gestation (mid gestation) and found that progesterone decreased after infection, possible due to the damage caused by the parasite, but did not establish any relation between the hormone and a possible modulation of the immune response [32, 33].

In conclusion, this work further characterizes the peripheral and placental immune responses after *T. gondii* experimental infection at the three terms of gestation in sheep, adding detailed information on ovine immune response to a previous work [2]. Our results show that infection with this protozoan influences the peripheral and placental immune response and that it is mediated by a mixed of Th1 (IFN-γ and TNF-α), Th2 (IL4) and Treg (IL10) cytokines. They also show differences between placental and peripheral immune responses, regarding those cytokines evaluated, and how there could be differences, especially on IL10 and IL4 at the placenta, depending on the term of gestation when infected. These differences are most probably a consequence of several complex processes where various factors, including gestation, parasite and host, play an interrelated role that warrants further investigation.

## Supplementary information

**Additional file 1. Sequences of primers used for cytokine real-time PCR (qPCR) and standard curve data.** a NCBI accession numbers are for ovine cDNA sequences used in primer design. Primer annealing was also checked with the *Ovis aries* genomic DNA sequences of the chromosome 3 for IFN-γ, the chromosome 20 for TNF-α, the chromosome 5 for IL4, the chromosome 12 for IL10 and the chromosomes 14 and 24 for β-actin in NCBI database [29]. b Minimal coefficient of regression (*R*^*2*^) of standard curves for each PCR target in all batches of amplification, based on tenfold dilutions (10^−1^−10^−7^) of 10 ng/µL from plasmid stocks. Ct values increased linearly until the level of 10^−7^ dilution of all plasmids. c Standard curve slopes. Minimal and maximal values for slopes for each PCR target in all batches of amplification. d Inter-assay coefficient of variation. CV values indicate the maximum and minimum CVs of all points from standard curves for each PCR target run in this study. Subscript numbers indicate curve point for CV values. (*) Indicates primers annealing at intron splice junctions. No amplification products were detected when ovine genomic RNA free-DNA samples were tested with cytokine primers (data not shown). All sequences of primers were previously described by Arranz-Solís et al. [14].

**Additional file 2. Comparison of average values of parasite burden and histological lesion at day 26 post-infection at the animals infected at the three terms of gestation: day 40 (G1), day 90 (G2) and das 120 (G3).** Table summarizing the result from the Castaño et al. [2] where the experimental design and clinical and lesional results from this experiment are detailed.

## Data Availability

The datasets generated during and/or analysed during the current study available from the corresponding author on reasonable request.
