# Supplementary material for: Peripheral and placental immune responses in sheep after experimental infection with Toxoplasma gondii at the three terms of gestation
Source: Vet Res. 2019 Sep 18;50:66. doi: 10.1186/s13567-019-0681-8 (PMC6751581; doi:10.1186/s13567-019-0681-8)
Supplement: Supplementary file 1 — Additional file 1. Sequences of primers used for cytokine real-time PCR (qPCR) and standard curve data. a NCBI accession numbers are for ovine cDNA sequences used in primer design. Primer annealing was also checked with the Ovis aries genomic DNA sequences of the chromosome 3 for IFN-γ, the chromosome 20 for TNF-α, the chromosome 5 for IL4, the chromosome 12 for IL10 and the chromosomes 14 and 24 for β-actin in NCBI database [29]. b Minimal coefficient of regression (R2) of standard curves for each PCR target in all batches of amplification, based on tenfold dilutions (10−1−10−7) of 10 ng/µL from plasmid stocks. Ct values increased linearly until the level of 10−7 dilution of all plasmids. c Standard curve slopes. Minimal and maximal values for slopes for each PCR target in all batches of amplification. d Inter-assay coefficient of variation. CV values indicate the maximum and minimum CVs of all points from standard curves for each PCR target run in this study. Subscript numbers indicate curve point for CV values. (*) Indicates primers annealing at intron splice junctions. No amplification products were detected when ovine genomic RNA free-DNA samples were tested with cytokine primers (data not shown). All sequences of primers were previously described by Arranz-Solís et al. [14]. [file 13567_2019_681_MOESM1_ESM.docx]

| **Target^a^** | **Primer** | **Primer sequences (5’-3’)** | **Product size (bp)** | ***R*^2 b^** | **Slope^c^** | **CV (%)^d^** |
| --- | --- | --- | --- | --- | --- | --- |
| **IFN-γ** (X52640.1) | QIFN-UP* | GATTCAAATTCCGGTGGATG | 110 | 0.996 | (-3.40) – (-3.20) | 2.26_(-6)_ – 1.68_(-2)_ |
|  | QIFN-RP | TTCTCTTCCGCTTTCTGAGG |  |  |  |  |
| **TNF-α** (NM_001024860.1) | QTNF-UP* | CCAGAGGGAAGAGCAGTCC | 126 | 0.996 | (-3.24) – (-3.19) | 6.69_(-2)_ – 2.28_(-7)_ |
|  | QTNFov-RP* | GGAGCGCTGATGTTGGCTAC |  |  |  |  |
| **IL4** (XM_004008636.1) | QIL4ov-UP* | CTGCCCCAAAGAACGCAACT | 154 | 0.996 | (-3.62) – (-3.48) | 7.24_(-1)_ - 0.95_(-6)_ |
|  | QIL4-RP* | TCATTCACAGAACAGGTCTTGCTT |  |  |  |  |
| **IL10** (NM_001009327.1) | QIL10-UP* | TGCTGGATGACTTTAAGGGTTACC | 60 | 0.997 | (-3.23) – (-3.22) | 7.22_(-1)_ – 2.33_(-7)_ |
|  | QIL10-RP | AAAACTGGATCATTTCCGACAAG |  |  |  |  |
| **β- actin** (NM_001009784.1) | BACTIN-UP* | ACACCGCAACCAGTTCGCCAT | 216 | 0,992 | (-3.63) – (-3.46) | 2.23_(-1)_-0.20_(-6)_ |
|  | QBACT216-RP | GTCAGGATGCCTCTCTTGCT |  |  |  |  |

**Additional file 1 Sequences of primers used for cytokine real-time PCR (qPCR) and standard curve data.**

^a^ NCBI accession numbers are for ovine cDNA sequences used in primer design. Primer annealing was also checked with the *Ovis aries* genomic DNA sequences of the chromosome 3 for IFN-γ, the chromosome 20 for TNF-α, the chromosome 5 for IL4, the chromosome 12 for IL10 and the chromosomes 14 and 24 for β-actin in NCBI database [30].

^b^ Minimal coefficient of regression (*R^2^*) of standard curves for each PCR target in all batches of amplification, based on 10-fold dilutions (10^−1^−10^−7^) of 10 ng/µL from plasmid stocks. Ct values increased linearly until the level of 10^-7^ dilution of all plasmids.

^c^ Standard curve slopes. Minimal and maximal values for slopes for each PCR target in all batches of amplification.

^d^ Inter-assay coefficient of variation. CV values indicate the maximum and minimum CVs of all points from standard curves for each PCR target run in this study. Subscript numbers indicate curve point for CV values.

(*) Indicates primers annealing at intron splice junctions. No amplification products were detected when ovine genomic RNA free-DNA samples were tested with cytokine primers (data not shown).

All sequences of primers were previously described by Arranz-Solís et al. [14].
