# Supplementary material for: Peripheral and placental immune responses in sheep after experimental infection with Toxoplasma gondii at the three terms of gestation
Source: Vet Res. 2019 Sep 18;50:66. doi: 10.1186/s13567-019-0681-8 (PMC6751581; doi:10.1186/s13567-019-0681-8)
Supplement: Supplementary file 2 — Additional file 2. Comparison of average values of parasite burden and histological lesion at day 26 post-infection at the animals infected at the three terms of gestation: day 40 (G1), day 90 (G2) and das 120 (G3). Table summarizing the result from the Castaño et al. [2] where the experimental design and clinical and lesional results from this experiment are detailed. [file 13567_2019_681_MOESM2_ESM.docx]

**Additional file 2**. **Comparison of average values of parasite burden and histological lesion at day 26 post infection at the animals infected at the three terms of gestation: day 40 (G1), day 90 (G2) and das 120 (G3).**

|  | Organ |  | Group | | |
| --- | --- | --- | --- | --- | --- |
|  |  |  |  |  |  |
|  |  |  | G1 | G2 | G3 |
| Parasite burden  ( tachyzoite number/mg ovine tissue) | Placenta |  | 5.49 (12.07^)a^ | 91.97 (1250.00) | NA |
|  | Brain |  | 5.38 (16.94) | 8.51 (118.20) | 0.30 (4.21) |
|  | Liver |  | 117.50 (395.40) | 13.49 (60.90) | 0 (69.96) |
|  |  |  |  |  |  |
| Histological lesion  (percentage of studied area showing lesion) | Placenta |  | 0.01^b^ | 0.03 | NA |
|  | Brain |  | 0.08 | 0.31 | 0.02 |
|  | Liver |  | 0.2 | 0.58 | 0.23 |
| ^a^ Medain (interquartile range) ;^b^ % of lesion in studied area | | | | | |
